# Nephrotic syndrome associated with primary atypical hemolytic uremic syndrome

**DOI:** 10.1590/2175-8239-JBN-2020-0050

**Published:** 2020-08-10

**Authors:** Diana Carolina Bello-Marquez, John Fredy Nieto-Rios, Lina Maria Serna-Higuita, Alfonso Jose Gonzalez-Vergara

**Affiliations:** 1 Universidad de Antioquia, Medellin, Colombia.; 2 Hospital Pablo Tobon Uribe, Medellin, Antioquia, Colombia.; 3 University of Tübingen, Institute of Clinical Epidemiology and Applied Biometrics, Tubingen, Germany.; 4 University Corporation Antonio Jose de Sucre, Sincelejo, Sucre, Colombia.

**Keywords:** Atypical Hemolytic Uremic Syndrome, Nephrotic Syndrome, Acute Renal Injury, Hypertension, Complement System Proteins, Síndrome Hemolítico-Urêmica Atípica, Síndrome Nefrótica, Lesão Renal Aguda, Hipertensão, Proteínas do Sistema Complemento

## Abstract

Primary atypical hemolytic-uremic syndrome is a rare disease characterized by non-immune microangiopathic hemolytic anemia, thrombocytopenia, and renal dysfunction; it is related to alterations in the regulation of the alternative pathway of complement due to genetic mutations. The association with nephrotic syndrome is unusual. We present here a pediatric patient diagnosed with primary atypical hemolytic-uremic syndrome associated with nephrotic syndrome who responded to eculizumab treatment.

## Resumo

A síndrome hemolítico-urêmica atípica primária é uma doença rara, caracterizada por anemia hemolítica microangiopática não-imune, trombocitopenia e disfunção renal; está relacionado a alterações na regulação da via alternativa do complemento devido a mutações genéticas. A associação com a síndrome nefrótica é incomum. Apresentamos aqui um paciente pediátrico com diagnóstico de síndrome hemolítico-urêmica atípica primária associada à síndrome nefrótica que respondeu ao tratamento com eculizumab.

## Introduction

Primary atypical hemolytic uremic syndrome (aHUS) is a rare disease caused by mutations that promote uncontrolled activation of the alternative complement pathway. It is associated with certain triggers leading to thrombotic microangiopathy (TMA) with multi-systemic compromise especially renal features^1^, but it is rarely associated with nephrotic syndrome. It is important to provide specific early treatment in these patients to reduce the associated high morbidity and mortality. We present here a case of a pediatric patient with aHUS-associated nephrotic syndrome who was treated with eculizumab and showed adequate response to treatment.

## Clinical Case

This was a 4-year-old child who was previously healthy and who had consulted for 12 hours of evolution of generalized edema and oliguria; he had an episode of viral rhinopharyngitis 7 days before, without fever, skin lesions, or other symptoms. Upon physical examination, he was found to have anasarca with a weight of 19 kg, height of 115 cm, blood pressure of 140/75 mmHg, respiratory rate of 22 resp/min, and afebrile. Laboratory tests documented severe anemia with hemoglobin of 6.6 g/dL, thrombocytopenia (platelets 60,200 x mm^3^), proteinuria (55 mg/m^2^/hour), and acute renal injury (creatinine 1.7 mg/dL). In addition, there was evidence of hypoalbuminemia (albumin: 1.8 mg/dL), hyperlipidemia, elevated Lactate dehydrogenase (LDH: 2,082 U/L), reticulocytosis, and schistocytes in peripheral blood. He was negative for direct Coombs test and had decreased haptoglobin. Serology extension studies were negative for Human immunodeficiency virus, hepatitis B virus, hepatitis C virus, and syphilis; C3 complement was 115 mg/dL and C4 complement was 38 mg/dL. He was negative for antinuclear antibodies (ANAs), anti-neutrophilic cytoplasmic autoantibodies (ANCAs), and anti-cardiolipin antibodies. Renal ultrasound showed a loss of bilateral echogenicity.

The diagnosis of aHUS with associated nephrotic syndrome was established. While the indication for receiving eculizumab was fulfilled, he was vaccinated against meningococcus. He began support management with red blood cells and received prophylactic oral penicillin. After 48 hours, he presented clinical deterioration with a decrease in hemoglobin values to 4.4 mg/dL, increased thrombocytopenia, progression of acute renal injury to KDIGO 3, a generalized tonic-clonic seizure, and worsening of arterial hypertension (154/92 mmHg). He was transferred to the intensive care unit where 20 mL/kg/day of plasma infusion was administered over 2 days. Peritoneal dialysis was initiated with transfusion support. Disintegrin and metalloprotease levels with ThromboSpondin type 1 motif (ADAMTS 13) values were normal (78%), and Shiga toxin in the fecal matter was negative. Six days after admission, 600 mg Eculizumab was started weekly for 3 weeks, and then every 14 days. This allowed the disease to be controlled without new TMA events. Renal replacement therapy was suspended 8 days after being initiated, and resolution of the nephrotic syndrome was obtained 8 weeks later without the use of steroids (proteinuria 3 mg/m^2^/hour, albumin: 4.3 g/dL). Subsequently, a genetic study was carried out (CENTOGENE laboratory) and reported a mutation of the CFI gene heterozygous variant c.1270A> C p. (Ile424Leu) and the ADAMTS 13 heterozygous variant c.559G> C p. (Asp187His). In addition, a MLPA test showed heterozygous deletion of the CFHR3 and CFHR1 genes.

**Figure 1:**
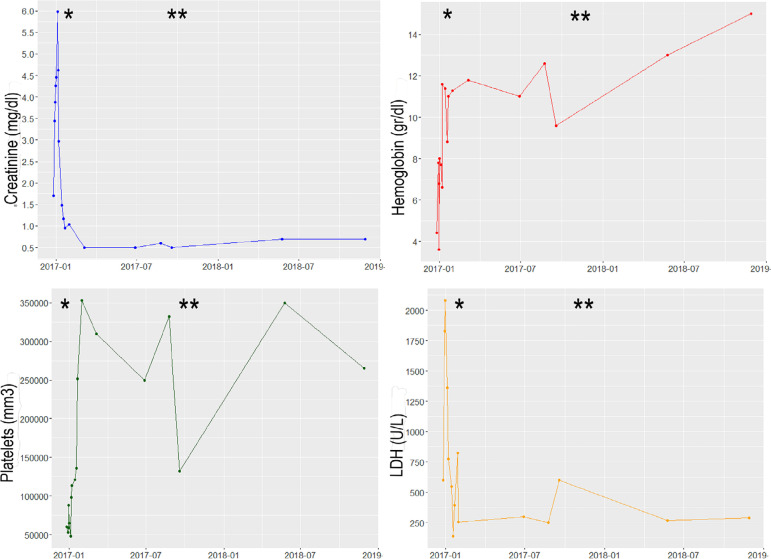
Laboratory values during follow-up.

**Figure 2:**
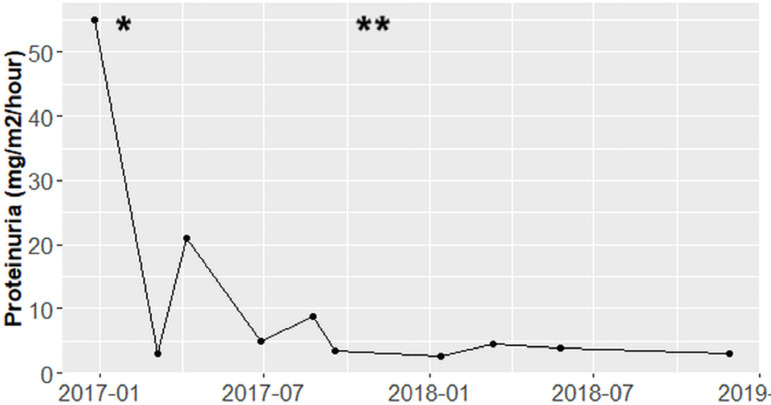
Proteinuria values (mg/m^2^/hour) during follow-up.

Chest radiographs showed findings compatible with pulmonary edema and cardiomegaly eight months after the patient was hospitalized due to respiratory distress and arterial hypertension. The echocardiogram showed an ejection fraction of 40% consistent with acute heart failure. The LDH increased to 600 U/L with anemia (Hb: 9.6 g/dL) and thrombocytopenia (platelets 132,000 mm^3^) without deterioration of renal function; infectious causes were ruled out. It was decided to increase the dose of Eculizumab to 600 mg every 14 days achieving control of the disease. After two years of follow-up, he continues in treatment with Eculizumab with normal renal function and in remission of the nephrotic syndrome (proteinuria 2.5 mg/m^2^/h, creatinine 0.6 mg/dL, cholesterol 150 mg/dL, triglycerides 94 mg/dL, and albumin 4.3 mg/dL) (Figure 1 and 2). There are no signs of heart failure, anemia, or thrombocytopenia (Figure 1). However, he still persists with arterial hypertension, which is managed with enalapril and hydrochlorothiazide.

*Started treatment with Eculizumab, **Increase in dose of Eculizumab

*Started treatment with Eculizumab, **Increase in dose of Eculizumab

## Discussion

We report a case of a patient with simultaneous diagnoses of nephrotic syndrome and aHUS with an adequate response to eculizumab therapy and no need to administer steroids; renal, hematological, cardiovascular, and central nervous system remission were achieved.

aHUS is a rare disease that belongs to the TMA spectrum^1^ and is characterized by a triad of microangiopathic hemolytic anemia with direct negative Coombs, thrombocytopenia, and acute renal injury. It is secondary to uncontrolled activation of the alternate pathway of the complement cascade due to genetic defects, which can be identified in more than 50% of cases^2^^-^ 4. Its differential diagnosis includes thrombotic thrombocytopenic purpura (TTP) due to deficiency in ADAMTS13 enzyme activity; hemolytic uremic syndrome associated with Shiga-toxin-producing bacteria (typical SHU), and secondary TMA^1^^,^ 3 ^,^ 5 ^,^ 6.

aHUS usually occurs abruptly and affects kidneys and/or multiple organs such as the brain, lungs, heart, gastrointestinal tract, etc. Renal involvement is evidenced by azotemia, arterial hypertension, hematuria, and/or proteinuria; it is very rarely associated with nephrotic syndrome^2^. In terms of therapy, the following options are currently available for the treatment of aHUS:

A) Plasma exchange at a dose of 1.5 plasma volumes per session with frozen fresh plasma. This was the treatment of choice until 2011 and it removes mutated complement factors and auto-antibodies to provide normal complement factors and promote hematologic remission. Such treatment has no significant improvement in renal function and has high morbidity and mortality at one-year follow-up. The main limitation is a high relapse rate, and it may be technically difficult to perform in a pediatric population^3^.

B) Plasma infusion at a dose of 10-20 mL/kg/day for 5-15 days and then five times a week for two weeks, then three times a week for two weeks, and finally, maintenance every 2-4 weeks. The usefulness of this therapy is based on the contribution of non-mutated complement factors^3^; however, it does not modify the course of the disease. Thus, it is considered as an alternative only when plasma exchange therapy is not available.

C) Eculizumab is a humanized anti-C5 monoclonal antibody that prevents the formation of the membrane attack complex by binding to human C5 with high affinity and preventing its cleavage by complement conversion into C5a and C5b (inhibition of terminal complement activity). 7 ^,^ 8. This medication induces both hematological and renal remission especially if it is started early (less than 7 days). It is considered a first line of treatment in children since 2011^8^.

D) Support measures include the use of renal replacement therapies, blood products, control of hypertension, and maintenance of acid-base balance, and electrolytes.

Nephrotic syndrome is a condition characterized by edema, hyperlipidemia, hypoalbuminemia, and proteinuria in the nephrotic range (>40 mg/m^2^/h)^9^^,^ 10. Complications associated with nephrotic syndrome include infections, cerebral venous thrombosis, pulmonary embolism, renal vein thrombosis, and acute renal injury. However, the presence of nephrotic syndrome is rare in the debut of a patient with aHUS^5^^,^ 11 as in the case reported here.

The literature contains 71 reported cases of patients with an initial diagnosis of glomerulopathies who subsequently developed HUS. They can be grouped into three different histological patterns according to the underlying pathology: nephrotic syndrome (17 patients), C3 glomerulonephritis/membranoproliferative glomerulonephritis (GNMP) (16 patients), and glomerulonephritis associated with vasculitis or mediated by immune complexes (32 patients). Of this series, two patients received treatment with eculizumab, the remainder received steroids, cyclophosphamide, cyclosporine, plasmapheresis, and rituximab. Of the total number of patients, eleven presented with chronic end-stage renal disease (ESRD), two persisted with proteinuria, and two died^2^.

From the pathophysiological point of view, proteinuria in the nephrotic range favors the appearance of thrombotic events due to an increase in thrombomodulin, release of von Willebrand factor, increase in platelet aggregation, and vascular endothelial growth factor deficit. In the contrary, aHUS induces greater proteinuria due to podocyte ischemia and their fusion as well as podocyte lysis due to activation of the membrane attack complex (C5-9); therefore, the presence of aHUS plus nephrotic proteinuria becomes a vicious circle with progressive deterioration of renal function^2^^,^ 12. It is not yet clear the role that glomerulopathies play in the development of primary aHUS; however, there is increasing evidence of the important relationship of these two entities especially those mediated by complement such as GNMP or C3 glomerulonephritis^13^.

The most frequent mutations in the pediatric population with aHUS are those of the CFH, CFI, and MCP genes. These are known from patients with CFH mutations who have more severe manifestations, worse prognosis, and greater risk of death or ESRD; 30% of these occurr during the first episode^3^, but this risk can increase up to 60% during the first year of evolution. Regarding CFI, although a rapid evolution to ESRD has been documented, more than 50% recover. In contrast, those with MCP mutations have a greater tendency to relapse, but few end up with ESRD^14^.

The genetic study of this patient documented the presence of mutations in the CFI, CFH, and ADMATS genes^13^. The first one corresponds to the CFI, which is a heterozygous variant c.1270A> C p. (Ile424Leu)-a variant that has been previously described as pathogenic for aHUS^14^ and has a higher frequency than expected for a rare disease. It has an uncertain significance according to the recommendations of the American College of Medical Genetics (ACMG). The heterozygous mutation of ADAMTS 13 c.559G> C p. (Asp187His) has previously been reported as pathogenic for congenital TTP in a patient in whom the disease was triggered by pregnancy^15^; however, the clinical findings in our patient were not compatible with TTP because the activity of the ADAMTS13 enzyme was completely normal. Finally, we used the MLPA test to detect a heterozygous deletion of the CFHR3 and CFHR1 genes that represent a greater risk for the development of aHUS; however, this deletion is very common in unaffected controls^15^^-^ 17; therefore, it is classified as a disease-associated variant according to the AMCG. Previous data have shown that 25% of patients with a mutation in CFI carried abnormalities in other complement genes that can increase the complete penetrance of the disease^18^.

In conclusion, we report a pediatric patient who simultaneously presented an aHUS and nephrotic syndrome with hematological, renal, neurological, and cardiovascular compromise; he was treated with Eculizumab with complete remission of systemic compromise without the need for use of steroids or other immunosuppressant drugs. To date (February 26, 2020), he remains on treatment without relapse. He is under strict medical surveillance.
